# Analysis of Minerals and Mineral Waters

**Published:** 1835

**Authors:** 


### VII.—Analysis of Minerals and Mineral Waters.

Professor Rogers, and Adjunct Professor Riddell, of the medical department of Cincinnati College, will be prepared in the ensuing winter, to analyze, with accuracy, any of our native minerals and medicinal waters that may be sent to them. With every mineral specimen there should be transmitted a specimen of the rock in which it was embedded; and mineral waters should be put up in sealed bottles, entirely filled, and corked under water.

